# Semantic Annotation, Representation and Linking of Survey Data

**DOI:** 10.1007/978-3-030-59833-4_4

**Published:** 2020-10-27

**Authors:** Felix Bensmann, Andrea Papenmeier, Dagmar Kern, Benjamin Zapilko, Stefan Dietze

**Affiliations:** 8 Linköping University, Linköping, Sweden; 9 University of Amsterdam, Amsterdam, Noord-Holland The Netherlands; 10 Department of Computer Science, Vrije Universiteit Amsterdam, Amsterdam, Noord-Holland The Netherlands; 11 St. Pölten University of Applied Sciences, St. Pölten, Austria; 12 FIZ Karlsruhe – Leibniz Institute for, Karlsruhe, Germany; 13 Karlsruhe Institute of Technology, Karlsruhe, Germany; 14 UAS St. Pölten, St. Pölten, Niederösterreich Austria; 15 Vienna University of Economics and Business, Vienna, Wien Austria; 16 VU Amsterdam, Amsterdam, The Netherlands; 17 ADAPT Centre, Trinity College Dublin, Dublin, Ireland; 18 GESIS - Leibniz Institute for the Social Sciences, 50667 Cologne, Germany; 19 Heinrich-Heine-University Düsseldorf, 40225 Düsseldorf, Germany; 20 L3S Research Center, 30167 Hannover, Germany

**Keywords:** Question feature extraction, Social sciences survey data, Semantic data modelling, Natural language processing

## Abstract

Semantic technologies offer significant potential for improving data search applications. Ongoing work thrives to equip data catalogs with new semantic search features to supplement existing keyword search and browsing capabilities. In particular within the social sciences, searching and reusing data is essential to foster efficient research. In this paper, we introduce an approach and experimental results aimed at improving interoperability and findability of social sciences survey items. Our contributions include a conceptual model for semantically representing survey items and questions, detailing meaningful dimensions of items, as well as experimental results geared towards the automated prediction of such item features using state-of-the-art machine learning models. Dimensions of interest include, for instance, references to geolocation and time periods or the scope and style of particular questions. We define classification tasks using neural and traditional machine learning models combined with sentence structure features. Applications of our work include semantic and faceted search for questions as part of our GESIS Search. We also provide the lifted data as a knowledge graph via a SPARQL endpoint for further reuse and sharing.

## Introduction

In the social sciences, questionnaire-based survey programs are the instrument of choice to collect information from a particular population. This survey data usually comprises attitudes, behaviours and factual information. To collect survey data, a research team usually composes a dedicated questionnaire for a population group and collects the data in personal interviews, telephone interviews, or online surveys. As this process is very complex and time-consuming, social scientists have a strong need for re-using both actual survey results for secondary analysis [8] as well as well-designed and constructed survey items, e.g. specific questions. In Germany, GESIS - Leibniz Institute for the Social Sciences^1^ is a major data provider that gathers, archives and provides survey data to researchers from all over the world. Datasets are searchable through GESIS Search^2^ or gesisDataSearch^3^. Current research on social scientists’ information needs indicates an increasing need for re-using survey data [17] and ongoing work already focuses on improving search applications with semantics e.g. from the users’ perspective [12].

A crucial factor in the process of finding and identifying relevant survey data is the quality of available metadata. Metadata includes general information like title, date of collection, primary investigators, or sample, but also more specific information about the study’s content like an abstract, topic classifications and keywords. So far, these metadata help to find a study of interest but they are less helpful if a researcher is interested in finding specific questions or variables. While a question is the text that is used to collect answers, variables contain the expression of the answers’ characteristics. For example, the fictitious question “What is your attitude towards the European Union?” has the variable “AttiduteEU” which could have the characteristics (1) negative, (2) neutral, or (3) positive. Currently, no dedicated vocabularies are used for capturing the semantics of variables or items, for instance, their scope, nature or georeferences (EU in this case).

A common way for researchers to find variables and questions is to first find suitable datasets. In a second step, they read exhaustive documentation to find concrete questions or variables that fit their research question. For comparing variables and finding similar variables, this process has to be repeated. Recently introduced variable search systems^4^ address this issue by providing a way to search for questions and variables with a common text-based search approach. However, the intention of a question or the concept to be measured are often not directly verbalized in its textual description.

In this paper, we examine how a variable’s content can be described more expressively, using state-of-the-art semantic technologies. Therefore, we focus on extracting and representing additional information from a question that go beyond tagging the questions with keywords and topics. Our approach is based on the ofness and aboutness concept of survey data introduced by [10]. While ofness refers to the literal question wording, which often reveals information about the topic of a question, the aboutness relates to the latent content. In our work, we focus on the aboutness aspects. This includes, for instance, the scope and nature of a question, e.g. whether the question asks for opinions or about a fact about the interviewee’s life.

The so called question features are designed to complement each other and are formally modeled as RDF(S) data model. We also introduce experiments for supervised classification models able to automatically predict question features. As the focus of our work is more on the question features and their systematic we started with established classifiers leaving more recent approaches for future work. Experiments are conducted on a real-world corpus of frequently used survey questions, consisting of 6500 distinct questions. For each question, we extract the question features by using a variety of text classification approaches, e.g., neural networks like LSTM. In addition, we generated a knowledge graph (KG) and publish the results via a dedicated SPARQL endpoint^5^.

Our main contributions can be summarized as followed: (1) We provide a taxonomy of question features and (2) a comprehensive data model describing the questions and the features in relation to each other. Finally, (3) we provide methods and first results for the prediction of one question feature, i.e. for populating a knowledge base of expressive question metadata.

The paper is structured as follows. First, we provide the related work in Sect. 2 and elaborate the design of the question features and the data model in Sect. 3. Afterwards, in Sect. 4, we describe our experiments on extracting the “Information type” question feature before we eventually close discussing application scenarios and draw a conclusion (Sect. 5).

## Related Work

In this section, we discuss related work, including available survey data catalog systems, relevant RDF vocabularies for model design and methods for feature extraction.

Some notable providers of social science survey data in Germany and internationally are GESIS, LifBi(NEPS)^6^, SOEP/DIW^7^, pairfam^8^ and ICPSR. These institutions allow their customers access to data and documentation on different levels. Smaller institutions are known for a narrow set of datasets, they do not host complex online catalogs but provide study documentation as HTML or PDFs online. However, sometimes they cooperate with larger institutions or consortiums that host their datasets. SOEP and pairfam, for example, take part in panaldata.org a data catalog for variables, questions, concepts, publications and topics. It provides text based search. Larger institutions like GESIS and ICPSR host large catalogs for study level data and sub-studylevel data. GESIS’ GESIS Search and ICPSR’s data portal are two examples for more complete search applications. Yet, to our knowledge there is no example of a variable catalog system that uses expressive and formally represented question features like the ones presented in this paper.

For our data model, we investigated related RDF vocabularies. [13] outlines best practices to consider when publishing data as Linked Open Data by e.g. reusing established vocabularies. Relevant work is found in vocabularies describing scientific data e.g. the DDI RDF discovery vocabulary^9^ [2, 3]. It is based on the Data Documentation Initiative (DDI) metadata standard, which is an acknowledged standard to describe survey data in the social sciences. DataCube^10^ focuses on statistical data. Large cross-domain vocabularies of relevance include Schema.org^11^ and DBpedia^12^. Further candidates are upper-level vocabularies like DOLCE-Lite-Plus^13^, as they serve more general terms and are not focused at specific domains.

With respect to methodological work on classification of short text, e.g. for predicting question features, approaches include the ones surveyed by [1], where the authors provide a survey on text classification examples for different tasks like “News filtering and Organization”, “Document Organization and Retrieval”, “Opinion Mining” or “Email Classification and Spam Filtering” applying various approaches e.g. “Decision Trees”, “Pattern (Rule)-based Classifiers”, “SVM Classifiers” and many more. The authors elaborate also on the experimental setups and best practices. Similar work can also be found in [20]. The survey presented in [24] elaborates on the special aspects of short texts and popular work on classifiers using semantic analysis, ensemble short text classification etc. is introduced. In [5] the authors present an approach specialized for short text classification leveraging external knowledge and deep neural networks. A famous short text corpus and target of many classification/extraction tasks is Twitter^14^. Our work relates for example to the extraction of specific dimensions e.g. sentiments [21] or events [27]. While individual approaches certainly overlap with ours, as they work on (rather arbitrary) short texts, our setup leverages specifics of survey questions which allows to compose our question features in a systematic way so that they complement each others and serve a common goal, i.e. better performance in a search system.

## Semantic Features of Survey Questions

Before we introduce our taxonomy of question features, we give a closer description of survey questions.

### Survey Questions

**Fig. 1.**
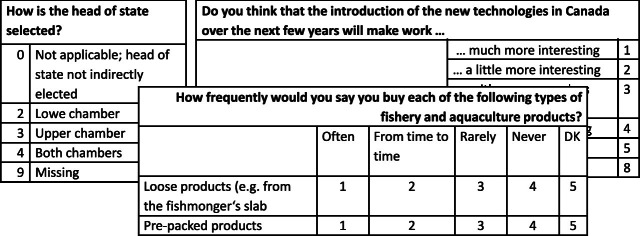
Example questions. CSES 2015 (left), ISSP 1997 (center) and Eurobarometer 2018 (right) [9, 16, 26]

A question in a questionnaire is described through a question text and predefined answer categories^15^. Figure 1 depicts three example questions. In some cases, when a group of questions differs in only the object they refer to, questionnaire designers assemble these in item batteries, where the items share question text and answer categories. An example can be seen in Fig. 1 (question in the center). A variable corresponds to either a complete question when there is only one answer available, or a question item. In the remainder of the paper and in our dataset, we treat questions having several items as separate instances and refer to them as “question-item pairs”. Questions without items are likewise a single question instance.

Survey questions are not necessarily questions in the grammatical sense, i.e. a single sentence with a question mark at the end. Many questions incorporate introductory texts and definitions for clarification. Additionally, they are often formulated as requests for the respondent or they are prompts for supplement. Meaning they are formulated as the first part of a statement, stopping with “...” and leaving the second part to the respondent to complete. The question instances in our dataset have between one and 171 words with 29 words on average.

Other properties documenting variables are an identifier, a label, interviewer instructions, keywords, topic classification, encoding in the dataset and more.

### A Taxonomy of Question Features

We assume that a search session for a question starts with a topic or keyword search and is subsequently refined through the use of facets. Our taxonomy presented in the following focuses on the facets. Therefor it does not include features regarding the actual topic which can be extracted by e.g. topic modelling. For our semantic description, we identified recurrent patterns in survey questions through literature [23], elaboration with domain experts as well as brainstorming. We looked into more than 500 questions and question-item pairs from over 200 studies. From this we compiled an initial list of potential question features to be discussed individually with two experts who we trust. Our foremost interest was to identify relevant filter criteria for social scientists. Subsequently, we oriented us along the requirements needed for use cases such as faceted search of items, questions or variables and identified some criteria any feature should adhere to. These include explicitness, distinctiveness, comprehensibility, a discrete value range (which may be described through a controlled vocabulary), meaningfulness, recurrence in our dataset, annotatability (practical^16^) and extractability.

We came up with a list of 11 question features involving features that describe the problem/task given to the respondent, e.g. the scene depicted, statements that can be made about the information asked, the tone and complexity of language or the nature of the object of the question.

Our features are presented in the following. The list names the question feature and provides a definition and the value range. For instance the question feature Time reference captures whether a question refers to the past, present or future of the respondents life, or whether a hypothetical scenario is depicted. Depending on the situation more than one value could be correct. I. e. the Information type was designed to be mutual exclusive. All question features are either of *- or 0..1- cardinality. The values are to be determined through individual approaches, e.g. a text classification or keyword matching, for example the value range for the question feature Geographic location is meant to correspond with the Geonames^17^ gazetteer. For reasons of conciseness, we omitted the definitions of the allowed values in the list. They are however presented online along with the KG documentation.

- **Information type.** The information type of a question characterizes which type of information the respondent is asked to state about the question object.
  Values: Evaluation (Sub-values: Willingness, Preference, Acceptance, Prediction, Assessment, Explanation), Fact (Sub-values: Demography, Participation, Activity, Decision, Use, Interaction, Behaviour, Life Events), Cognition (Sub-values: Emotion, Knowledge, Perception, Interest, Motivation, Believes, Understanding).
- **Focus.** This feature characterizes the focus of the question object. Whether it is focused towards the respondent, another person or if it is wide as in a general question. Values: Self focus, External focus (Sub-values: Family/Member of family, Acquaintance, Affiliate, Public Person, Institution, Object focus/item focus, Event focus), Generic/universal focus and Self+external focus.
- **Time reference.** Time reference characterizes the question’s time reference wrt. past, present and future. Values: Past, Present, Future, Hypothetical - past, Hypothetical - present, Hypothetical - future.
- **Periodicity.** Periodicity characterizes the duration and periodicity of the time the question refers to. Values: Point in time, Time span, Periodic point in time, Unspecific.
- **Information intimacy.** Information intimacy characterizes the sensitivity of the requested information with respect to personal life. Values: Private, Public.
- **Relative location.** The relative location states a location that is mentioned which is not described by a geographic name but by its meaning for the respondent. Values: Without, Apartment/Flat, Neighborhood/Street, Municipality/City, Region, Country, Continent, World, Place of work, Journey, Stays abroad.
- **Geographic location.** The name of a geographic location if mentioned. Values: <Continent>, <Countries>, <Region>, <Government region>, Others, Without, Unspecific, Mixed/Multiple
- **Knowledge specificity.** Describes the specificity of the knowledge that is required to answer the question according to the origin of that knowledge. Values: School, Daily life, Special knowledge.
- **Quantification.** This feature captures the quantification of the answer. As opposed to Information type it is more concrete and close to physical quantity. Values: Frequency, Date time, Time dimension, Spatial expansion, Mass, Amount, Level of agreement, Boolean, Rating, Naming/Denomination, Order, Comparative.
- **Language tone.** Language tone characterizes the degree of formality or tone that is applied in the question. Values: Colloquial language, Formal language, Jargon/technical language.
- **Language complexity.** Language complexity characterizes the complexity of phrasing applied in the question. Values: Simple language, Moderate language level, Raised language level.

### Data Model and Vocabulary

**Fig. 2.**
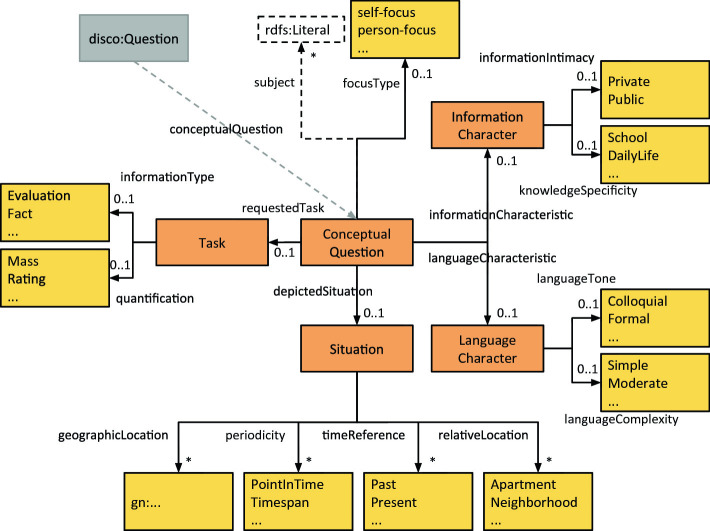
Data model. The arrows represent the question features and groups, yellow boxes indicate the value range for a question feature, orange boxes indicates a group. The gray and the white box help to connect to the context. (Color figure online)

Our model connects to the DDI-RDF Discovery vocabulary (DISCO) [2, 3]. It is an RDF representation of the Data Documentation Initiative (DDI) data model, an established standard for study metadata, maintained by the DDI Alliance^18^. While in DISCO the focus is set on a formal documentation of a questionnaire and its questions, our model extends the survey questions by a conceptual representation with the content dimensions (question features) described in the list above. We arranged the question features in groups for a better overview and to be able to link and reuse related and similar question characteristics in the future. When designing the model, we tried to identify terms in established vocabularies like those mentioned in related work in order to follow best practices and facilitate reuse and interpretation of the data. Since the scope of our model is specialised towards the social sciences, reflecting very particular dimensions and features, for a large number of classes and properties in our model no adequate terms could be found in existing vocabularies. In Fig. 2 we present the designed model on a conceptual level.

Our dataset is available online^19^ along with a SPARQL endpoint and webpage describing the data and providing example queries.

## Annotation and Enrichment

In total, there are 165 184 machine readable and sufficiently documented variables (i.e. questions or question-item pairs) available. The 101 554 variables having an English question text are included in our data set. To create a gold standard, we drew uniformly at random 6500 variables for manual annotation from this dataset. GESIS Search^20^ provides access to all studies and their documentations involved in our work.

### Manual Annotation

In a first step, we decided to focus on the feature Information type. We recruited an annotator based on annotation experience and knowledge about social science terminology to annotate this feature type. Before the annotation, the label categories were explained to the annotator. In a training phase with 100 question instances (excluded from the final data set), annotations that the annotator perceived as difficult were discussed with the authors.

The custom web interface guided the annotation process by displaying the question text, item text (if available), and the answer options. The annotator selected exactly two labels for each question, one label for Information type L1 and one label for L2. Once the annotator selected a label for L1, the corresponding sub-values (L2) are presented to reduce cognitive load and avoid mistakes. For each question instance, the annotator reported her level of confidence on a scale of 0 (“not confident at all”) to 10 (“very confident”). In total, 511 question instances were omitted due to an annotator-certainty of under 4. The final annotated dataset, therefore, consists of 5989 question instances.

At the end of the process, the annotator annotated 1200 question instances a second time to calculate the test-retest reliability. Cohen’s kappa coefficient reaches a substantial self-agreement of .72 for L1 and .64 for L2, a sufficient level of reliability to trust the consistency of the annotator.

### Automatic Prediction

Based on the provided annotations for the Information type, we can extract this question feature automatically from the natural language text of the question and the item text, if applicable. In our case, predicting the question features described in Sect. 3 represents a multi-class classification task. We tested and compared multiple classifiers on this task each for L1 and L2: LSTM [11], RandomForest [4], Multinomial Naive Bayes [18], Linear Support Vector Machines [7] and Logistic Regression [15]. We also took different kinds of input features into account: Word sequences and text structure.

**Fig. 3.**
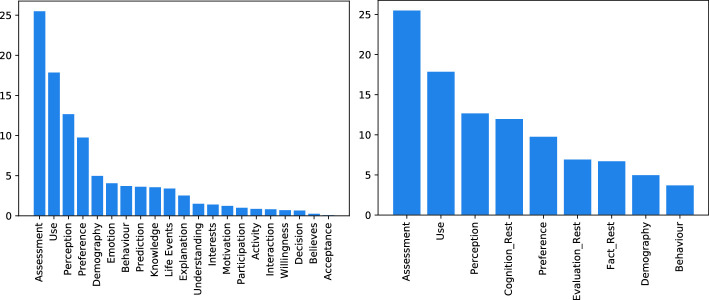
Distribution of Information type L2 labels, original (left) and merged (right)

The annotated values for L1 are distributed as follows: 42.08% Evaluation, 33.30% Fact and 24.62% Cognition. For L2, we provide the original distribution in Fig. 3 (left). The y-axis shows the percentages of relative occurrence. While the classes of Information type L1 are approximately balanced, the classes of Information type L2 are strongly imbalanced. We assume by experience that the amount of data points in the smaller classes of L2 (e.g. “Believes” with 15 instances, or “Decision” with 39 instances) is too low to train a classifier and therefore combine classes with insufficient instances into umbrella classes as shown in Fig. 3 (right). For each class in L1, there is an umbrella class in L2: “Fact_Rest” (combining “Participation”, “Activity”, “Decision” and “Life Events”), “Cognition_Rest” (combining “Emotion”, “Knowledge”, “Interest”, “Motivation”, “Believes” and “Understanding”) and “Evaluation_Rest” (combining “Willingness”, “Acceptance”, “Prediction” and “Explanation”). In the final set of classes for L2 there are nine labels: “Assessment”, “Use”, “Perception”, “Cognition_Rest”, “Preference”, “Evaluation_Rest”, “Fact_Rest”, “Demography”, “Behaviour”, with the biggest class (“Assessment”) having 1523 instances, and the smallest (“Behaviour”) containing 221 samples. The umbrella classes of L2 are currently not part of the data model (cf. Sect. 3.3) as the respective L1 class can be used instead e.g. “Cognition” for “Cognition_Rest”.

**Using Word Sequences.** As natural language can be understood as a sequence of words, modern sequence models are a good fit to classify natural languages. Long-Short Term Memory (LSTM) models have shown to outperform other sequential neural network architectures [11] when applied to context-free languages such as natural language. We therefore employ an LSTM architecture to classify the natural language questions in our data set and will subsequently refer to this approach as seq_lstm.

We implemented the LSTM network using Keras’ [6] sequential model in Python 3.6. The model has a three layer architecture, with an embeddings input layer (embeddings with dimension 100), an LSTM layer (100 nodes, dropout and recurrent dropout at 0.2), and a dense output layer with softmax activation. The model is trained with categorical cross-entropy loss and optimised on accuracy (equals micro-f1 in a single class classification task).

The embeddings layer uses the complete training data to compute word vectors with 100 dimensions. The question instances are preprocessed by removing all punctuation besides the apostrophe and converting all characters to lower case. For tokenisation, the texts are split on whitespaces. Since the input sequences to the embeddings layer need to be of equal length, we pad the sentences to a fixed length of 50 words by appending empty word tokens to the start of the sequence. On average, the question-item sequences contain 29 words, with a standard deviation of 16 words. Sequences longer than 50 words (8% of the question-item pairs, whereof 50% are shorter than 60 words) are cut off at the end to fit the fixed input length.

**Using Text Structure.** For this second approach, we used the structure of the question texts as input for our models. The idea behind this approach is the assumption of a dependency existing between the sentence structure of a question and the Information type.

Expecting the item text to provide valuable information for predicting the Information type through the text structure, we concatenated question text and item when an item was present. We extracted the structure from the otherwise unprocessed text by using a Part-of-speech (POS) parser to shallow parse (also referred to as light parsing or chunking) the question instances into a tree of typed chunks. From this we used the chunk types except for the leaf nodes (the POS tags) to define a feature vector where each component represents the number of occurrences of a specific chunk type. There are 27 different chunk types.

For the actual parsing we choose the Stanford PCFG parser in version 3.9.2 [19] as it is well-known and tolerant towards misspellings. However, some special cases in the phrasing introduce noise. Some expressions miss expressiveness as they refer to information presented in a previous question (“How is it in this case?”) or in the answer categories (“Would you ...”). Furthermore, misspellings and similar errors introduce additional noise. Since the parser was able to provide a structure for all samples we did not have to exclude any samples. Leaving all 5989 samples for use.

We started testing using standard classifiers RandomForest (str_rf), Multinomial Naive Bayes (str_mnb), Linear Support Vector Machines (str_svc) and Logistic Regression (str_logreg) from the scikit-learn [22] library for Python. For each model we performed grid hyperparameter tuning on the training set with 5-fold cross-validation. We report parameters deviating from the default configuration. For str_svc we used C=0.5, max_iter=5000 and ‘ovr’=multi_class mode. For str_rf n_estimators=200, max_features=3 and max_depth=50 was used. Again for str_logreg we applied C=10 and max_iter=5000. Finally str_mnb was used with alpha=3.

### Evaluation Setup

For evaluating, we employ five-folds cross-validation with 80% training and 20% test set split and use the manual annotations as ground truth. For the best performing approach for predicting Information types L1 and L2, we also present and discuss the confusion matrices.

### Results

Table 1 displays the results for the L1 and L2 Information types. The first column states the name of the respective approach and model. The following two columns contain micro-f1 and macro-f1 for the L1 Information types and the remaining two columns do the same for the L2 Information types.

**Table 1.** Results of L1 and L2 Information type extraction

Approach	L1		L2	
	micro-f1	macro-f1	micro-f1	macro-f1
seq_lstm	**0.7640**	**0.7455**	**0.4793**	**0.482**
str_svc	0.5437	0.524	0.3444	0.2610
str_rf	0.6287	0.5751	0.4578	0.3754
str_logreg	0.5454	0.5329	0.3386	0.2844
str_mnb	0.5305	0.526	0.3377	0.2656

As we can see in Table 1, L1 seq_lstm has the highest micro-f1 score with 0.7640 followed by the group of str_-approaches which range between 0.5305 and 0.6287. The macro-f1 follows the same pattern with seq_lstm at 0.7455 and the others again grouped together and more than 0.17 points beneath. This is similar for L2 where seq_lstm again has the best micro-f1 and macro-f1 scores at 0.4793 and 0.482.

Our anticipated usage scenario is a facetted search in a data search portal i.e. the GESIS Search. Here users will be presented the question features as facets and be allowed to use them to define their search request more precisely. Due to the infinite ways to formulate questions (and to specify classes), sometimes the assignment of a question to a class is ambiguous, also when done manually. Different users may associate a certain question with a different class and may still be correct. Thus, our intuition is that an F1 score of 0.7 could be counted as suitable.

**Fig. 4.**
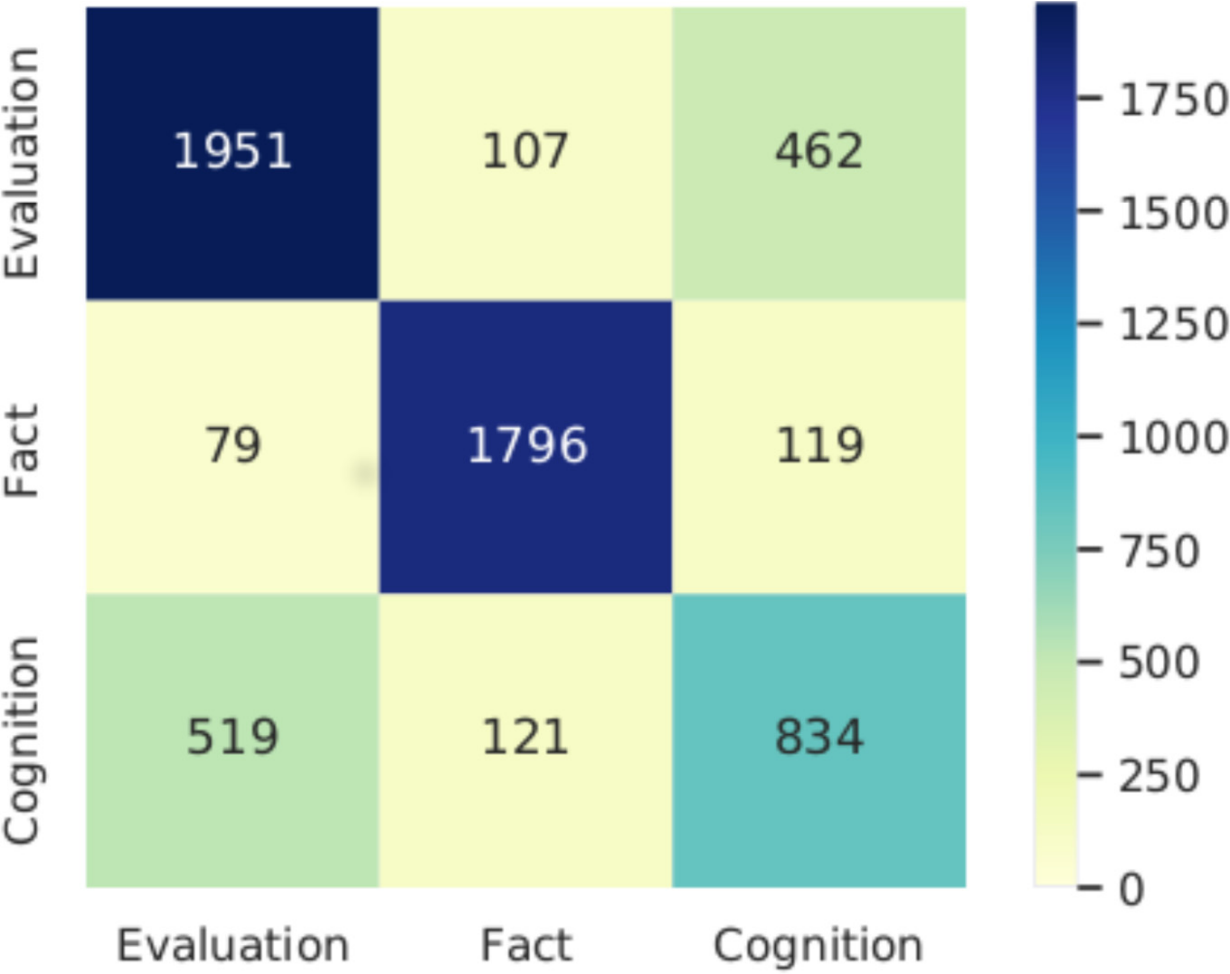
Confusion matrix for LSTM classifier on Information type L1

**Fig. 5.**
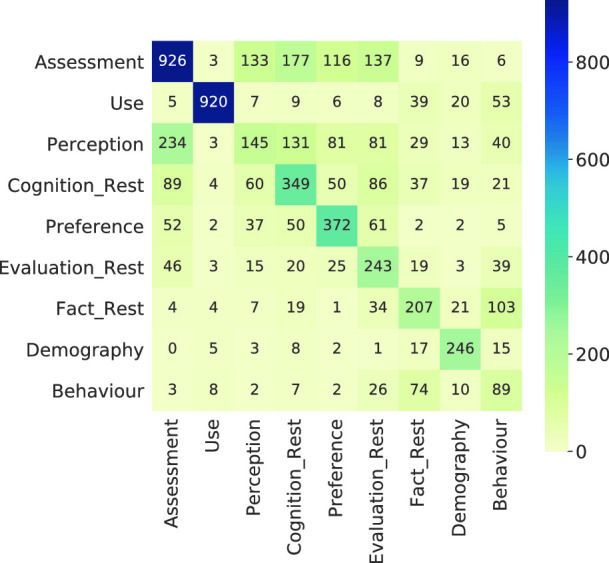
Confusion matrix for LSTM classifier on Information type L2

For L1 seq_lstm matches this goal. Also the str_-approaches are not out of range. However, results for L2 will need to be improved. Performance limiting factors may be low expressiveness of features and too similar classes. Given the high number of classes for L2 we are content with the models’ performances, however for the use case it might be better to merge some of the classes. For closer elaboration we present the confusion matrices in the Figs. 4 and 5.

In the diagrams, the predicted classes are on the X-axis and the actual classes are on the Y-axis. Both confusion matrices show little mispredictions of “Fact” or “Fact”-subclasses. In contrast, “Evaluation” and “Cognition” get confused more often. Especially in Fig. 5 “Assessment” (sub-class of “Evaluation”) gets mispredicted as “Perception” (sub-class of “Cognition”) and vice-versa. Also, a notable fraction of “Assessment” is confused with “Cognition_Rest”. Looking at the concerned classes’ labels, it is apparent that the concepts they represent are also for humans not easy to tell apart.

To test for this we conducted a small experiment for inter-annotator agreement where we reannotated 200 of the samples through two extra annotators. It resulted in an average Cohen’s $\kappa$ of 0.61 for L1 and 0.53 for L2 and Krippendorf’s $\alpha$ of 0.55 and 0.44. These values, except for $\kappa =0.61$, substantiate the notion that the task is even for humans not trivial. Which again indicates an indistinct design of the Information type classes, especially for L2. Supposedly a pilot study including multiple human annotators could help to define a clearer set of classes. However, classes should have intuitive denominations as complex artificial classes are hard to communicate to the users. Another way to overcome this could be to redesign the task as multi-class classification task. This however would come at the cost of simplicity for the user. Anyway, for this experiment the numbers show validity of our approach to a certain degree. An interesting question in this context will be to determine how the results change if the threshold for the confidence score for the inclusion of annotated questions into the dataset is raised.

A few things that could be improved are e.g. the selection of features for the str_-approaches which is rather sparse at the moment i.e. the feature vector might not carry enough information for the classifiers. Hence, a solution could be to extend the feature selection by the inclusion of signal words. E.g. “think”, “find”, “believe” may indicate opinions.

Once there are more question feature extractions available these can be used as input for each other leveraging potential interdependencies between then, e.g. in “Fact” questions certain values for “Quantification” might be more likely. Following the thought the test structure approaches could potentially be reused to extract some of the remaining question features directly, e.g. Language tone, Language complexity or Focus.

Str_* and seq_lstm approaches take different/complementary kinds of features into account. That is, str_* leverages solely the grammatical structure of a sentence, seq_lstm uses sequences of words. Thus, our intuition is that there is potential for a combination of them e.g. by using the predictions of both types of the classifiers as input into a meta-classifier. A closer analysis on the nature of mispredictions of the str_-classifiers will be conducted in this context.

## Conclusion

We present an approach to support the search of social science survey data by defining and implementing methods to annotate survey questions with semantic features. These dimensions complement existing topic and keyword extraction and allow for a finer grained semantic description.

We defined the dimensions as a taxonomy of question features (contribution 1), and designed a data model to describe the annotated data with the dimensions and lifted it together with the variable descriptions to RDF for re-use in other use-cases (contribution 2). Eventually, we examined approaches to predict the first question feature, the Information type, by means of classification tasks and present word sequences in combination with LSTM as a promising way (contribution 3). However, we consider combining it with one of the text structure approaches in the future.

Our question feature model offers many possibilities for applications. It is especially designed to be integrated in a facet filter scenario, but provides also multiple options for use in data linking, sharing and discovery scenarios. We target the GESIS Search https://search.gesis.org for a possible deployment. It is an integrated search system allowing search of multiple resource types including “Research data”, “Publications”, “Instruments & Tools”, “GESIS Webpages”, “GESIS Library” and “Variables & Questions”. The current filter offers the facets *year*, *source* and *study title* for the category of “Variables & Questions”. These will be complemented with our Information type feature. Besides lowering the assessment times for searchers per study, it could also improve re-use frequency and findability especially for less known datasets. Accordingly, less-experienced users may find it easier to orient themselves. Given that an already annotated training set can be reused, data providers in turn benefit from reduced efforts in variable documentation since this can be done automatically.

We are positive that there are additional use cases where a subset of our features can be reused to semantically describe textual contents. For example, short descriptions or titles of e.g. images can be annotated with the situation features. Also, language and knowledge features are applicable for these scenarios and can help to assess a text by getting to know the audience.

In future work, we plan to annotate and predict more features and fine tune the presented approach. Furthermore, a user study is planned to test for fitness in terms of (a) comprehensiveness of the facet and its values, (b) acceptance of the concept of the Information type and (c) trust in the accuracy of the annotation. A revision of the question feature design might still be necessary in order to fit user acceptance.

## Footnotes

1 https://www.gesis.org.

2 https://search.gesis.org/.

3 https://datasearch.gesis.org/start.

4 like ICPSR https://www.icpsr.umich.edu [25], GESIS Search https://search.gesis.org [14], paneldata.org https://paneldata.org/.

5 http://data.gesis.org/questionfeaturessample/site.

6 https://www.neps-data.de/Mainpage.

7 https://www.diw.de/en/soep.

8 https://www.pairfam.de/en/.

9 http://rdf-vocabulary.ddialliance.org/discovery.html.

10 https://www.w3.org/TR/vocab-data-cube/.

11 http://schema.org.

12 http://dbpedia.org/ontology/.

13 https://www.w3.org/2001/sw/BestPractices/WNET/DLP3941_daml.html.

14 https://twitter.com.

15 We do not consider open questions at the moment, as there are none in the data.

16 A human annotator needs to be able to work out the correct annotation with reasonable effort. E.g., long or nested value ranges, rare and too specific or too similar terms need to be avoided.

17 https://www.geonames.org/.

18 https://ddialliance.org/.

19 http://data.gesis.org/questionfeaturessample/site.

20 https://search.gesis.org, category research data.
